# Impacts of chest compression cycle length and real-time feedback with a CPRmeter® on chest compression quality in out-of-hospital cardiac arrest: study protocol for a multicenter randomized controlled factorial plan trial

**DOI:** 10.1186/s13063-020-04536-3

**Published:** 2020-07-08

**Authors:** Clément Buléon, Jean-Jacques Parienti, Elodie Morilland-Lecoq, Laurent Halbout, Eric Cesaréo, Pierre-Yves Dubien, Benoit Jardel, Christophe Boyer, Kévin Husson, Florian Andriamirado, Xavier Benet, Emmanuel Morel-Marechal, Antoine Aubrion, Catalin Muntean, Erwan Dupire, Eric Roupie, Hervé Hubert, Christian Vilhelm, Pierre-Yves Gueugniaud, on behalf of the CILICA-HS study group

**Affiliations:** 1 UNICAEN, CHU de Caen Normandie, Pôle Réanimations-Anesthésie-SAMU, Normandie University, 14000 Caen, France; 2 UNICAEN, CHU de Caen Normandie, Unité de Biostatistiques et de Recherche Clinique, Normandie University, 14000 Caen, France; 3 Department of Emergency Medicine, SAMU 69, Hospital Edouard Herriot, University Hospital of Lyon, Lyon, France; 4 Department of Anaesthesiology and Intensive Care, SAMU 76, Rouen University Hospital, Rouen Cedex, France; 5 SAMU Amiens, CHU Amiens-Picardie, Amiens, France; 6 Emergency Medicine Department and SAMU 59, Lille University Hospital, Lille, France; 7 Emergency Department, Centre Hospitalier d’Evreux, Evreux, France; 8 Emergency Department, Centre Hospitalier du Havre, Le Havre, France; 9 Emergency Department, Centre Hospitalier d’Elbeuf Louviers Val-de-Reuil, Elbeuf, France; 10 Emergency Department, Centre Hospitalier de Lisieux, Lisieux, France; 11 Emergency Department, Centre Hospitalier de Cherbourg, Cherbourg, France; 12 Emergency Department, Centre Hospitalier de Valenciennes, Valenciennes, France; 13 University Lille, EA 2694 - Santé Publique: Épidémiologie et Qualité des Soins, F-59000 Lille, France; 14 French National Out-of-Hospital Cardiac Arrest Registry Research Group, Registre Électronique des Arrêts Cardiaques, Lille, France

**Keywords:** Cardiac arrest, Cardiopulmonary resuscitation, Chest compression, Quality, Chest compression fraction, No-flow, Guidance

## Abstract

**Background:**

With a survival rate of 6 to 11%, out-of-hospital cardiac arrest (OHCA) remains a healthcare challenge with room for improvement in morbidity and mortality. The guidelines emphasize the highest possible quality of cardiopulmonary resuscitation (CPR) and chest compressions (CC). It is essential to minimize CC interruptions, and therefore increase the chest compression fraction (CCF), as this is an independent factor for survival. Survival is significantly and positively correlated with the suitability of CCF targets, CC frequency, CC depth, and brief predefibrillation pause. CC guidance improves adherence to recommendations and allows closer alignment with the CC objectives. The possibility of improving CCF by lengthening the time between two CC relays and the effect of real-time feedback on the quality of the CC must be investigated.

**Methods:**

Using a 2 × 2 factorial design in a multicenter randomized trial, two hypotheses will be tested simultaneously: (i) a 4-min relay rhythm improves the CCF (reducing the no-flow time) compared to the currently recommended 2-min relay rate, and (ii) a guiding tool improves the quality of CC. Primary outcomes (i) CCF and (ii) correct compression score will be recorded by a real-time feedback device. Five hundred adult nontraumatic OHCAs will be included over 2 years. Patients will be randomized in a 1:1:1:1 distribution receiving advanced CPR as follows: 2-min blind, 2 min with guidance, 4-min blind, or 4 min with guidance. Secondary outcomes are the depth, frequency, and release of CC; length (care, no-flow, and low-flow); rate of return of spontaneous circulation; characteristics of advanced CPR; survival at hospital admission; survival and neurological state on days 1 and 30 (or intensive care discharge); and dosage of neuron-specific enolase on days 1 and 3.

**Discussion:**

This study will contribute to assessing the impact of real-time feedback on CC quality in practical conditions of OHCA resuscitation. It will also provide insight into the feasibility of extending the relay rhythm between two rescuers from the currently recommended 2 to 4 min.

**Trial registration:**

ClinicalTrials.gov, NCT03817892. Registered on 28 January 2019

## Background

Out-of-hospital cardiac arrest (OHCA) remains a challenge for prehospital rescue. With an incidence between 5 and 15 per 10,000 and a survival rate of only 6 to 11% [1–6], there is still room for improvement in care to reduce the morbidity and mortality of these patients. The quality of cardiopulmonary resuscitation (CPR) is at the heart of the last three 5-year recommendations [7–9]. The latest recommendations emphasize the importance of professionals applying the highest possible quality of CPR and chest compressions (CC) [9].

The ratio of the time during which the CC are performed (low-flow) to the total time of resuscitation is referred to as the chest compression fraction (CCF). During CPR, minimizing CC interruptions, and therefore increasing the CCF, is essential, as this is an independent factor of cardiac arrest (CA) survival [10, 11]. CC interruptions are deleterious in at least three ways. First, they are a source of direct stoppage of cerebral and coronary perfusions, potentially altering the neurological prognosis and the probability of return of spontaneous circulation (ROSC) [12]. Second, the quality of the cardiac output generated by CC drops when resuming after an interruption of more than 30 s, the cutoff below which several CC can restore the best cardiac output possible [12, 13]. Third, CC interruptions automatically decrease the CC rate per minute, and difficulty in reaching the upper target of the guidelines’ CC rate has been linked to a significantly higher ratio of ROSC [14]. Reducing these interruptions and improving CC is therefore a major goal of improving CPR. The recommendations state that the CCF must be greater than 60%, and some experts estimate that a CCF of 80% is possible [15, 16].

The outcome of out-of-hospital cardiac arrest (OHCA) is significantly, positively, and independently correlated with the suitability for different CCF targets, CC frequency, CC depth, and brief predefibrillation pause (< 10 s) [17, 18]. Mechanical CC devices have not proven their superiority over manual CC [19], and manual CC remains the gold standard. There is evidence that CC guidance improves adequacy to recommendations and allows closer alignment with the CC frequency, depth, and release objectives [20]. We have demonstrated in simulation that the guidance of the CC delays the deterioration of the overall quality of the CC and its components (frequency, depth, and release) related to fatigue during an extended CC beyond the 2-min CC relay currently recommended [21].

Strategies to better match the recommendations regarding the quality of the CC associated with an improvement in CC should add or even enhance their beneficial effects for the management of CA. Achieving high-quality CPR requires the measurement of the CPR quality (CC and CCF) [22, 23].

This idea of a strategy of support enhanced by the “bundle” of concepts is developing in the literature. Thus, Cheskes et al. [24] describe a “high-quality CPR” such as the combination of a CCF greater than 70% and reaching the objectives in the recommendations for frequency and depth of CC.

The use of tools guiding CC quality still needs to be specified. Indeed, studies on their use in real-life situations are criticized for their methodological qualities and their sample sizes [25]. The use of a real-time guidance tool is proposed as an option in the latest recommendations without being mandatory due to a lack of current evidence [9]. Its use or nonuse does not imply any obvious loss of success for the patients. Evidence of its usefulness therefore remains to be sought.

For this reason, we want to perform an original, randomized, multicenter study to provide some answers to questions about the possibility of improving CCF by lengthening the time between two CC relays and the effect of guidance on the quality of the CC.

The design of the study will also allow us to investigate a possible combined effect of CC guidance and CC relay timing. The duration of a 2-min CC cycle between the two currently recommended relays does not have a solid evidence-based rationale and corresponds to a duration for which the CC effort can a priori be maintained while retaining efficiency [9, 26]. Objective measures have shown that the quality of the CC can be sustained beyond 2 min. Increasing the duration of a CC cycle could reduce the number of CC interruptions and thus improve the CCF.

We therefore formulate two hypotheses that we will test simultaneously using a 2 × 2 factorial design in a multicenter, randomized trial. The first assumption is that a 4-min relay rhythm improves the CCF (reducing the no-flow time) compared to the currently recommended 2-min relay rate. The second hypothesis is that a guiding tool improves the quality of CC.

The CPRmeter^®^ (guidance tool used in this study) will record data on the CC and their quality (depth, frequency, release, CPRmeter^®^ use time, no-flow time, and low-no-flow time) and will provide real-time feedback on CC for the guided group (the other group—blind—will have the screen masked by a screen cap).

Over a period of 2 years, this study will include 500 adult patients presenting with a nontraumatic OHCA for which advanced CPR is undertaken. We hope to improve the knowledge on the optimal rhythm of the CC relay and to validate “in vivo” the value of the guidance attained on manikins. This study should clarify the recommendations with a high level of evidence in this area and thus contribute to improving the prognosis of victims of OHCA.

### Trial objectives

The two main objectives of the 2 × 2 factorial plan are as follows:

- Objective 1: To determine whether the CCF gained from the CC relay rhythm of 4 min or 2 min is superior
- Objective 2: To determine whether the quality of the CC, as measured by correct compression score (CCS), is superior with guidance or with blinding

## Methods

### Study design and settings

**Fig. 1:**
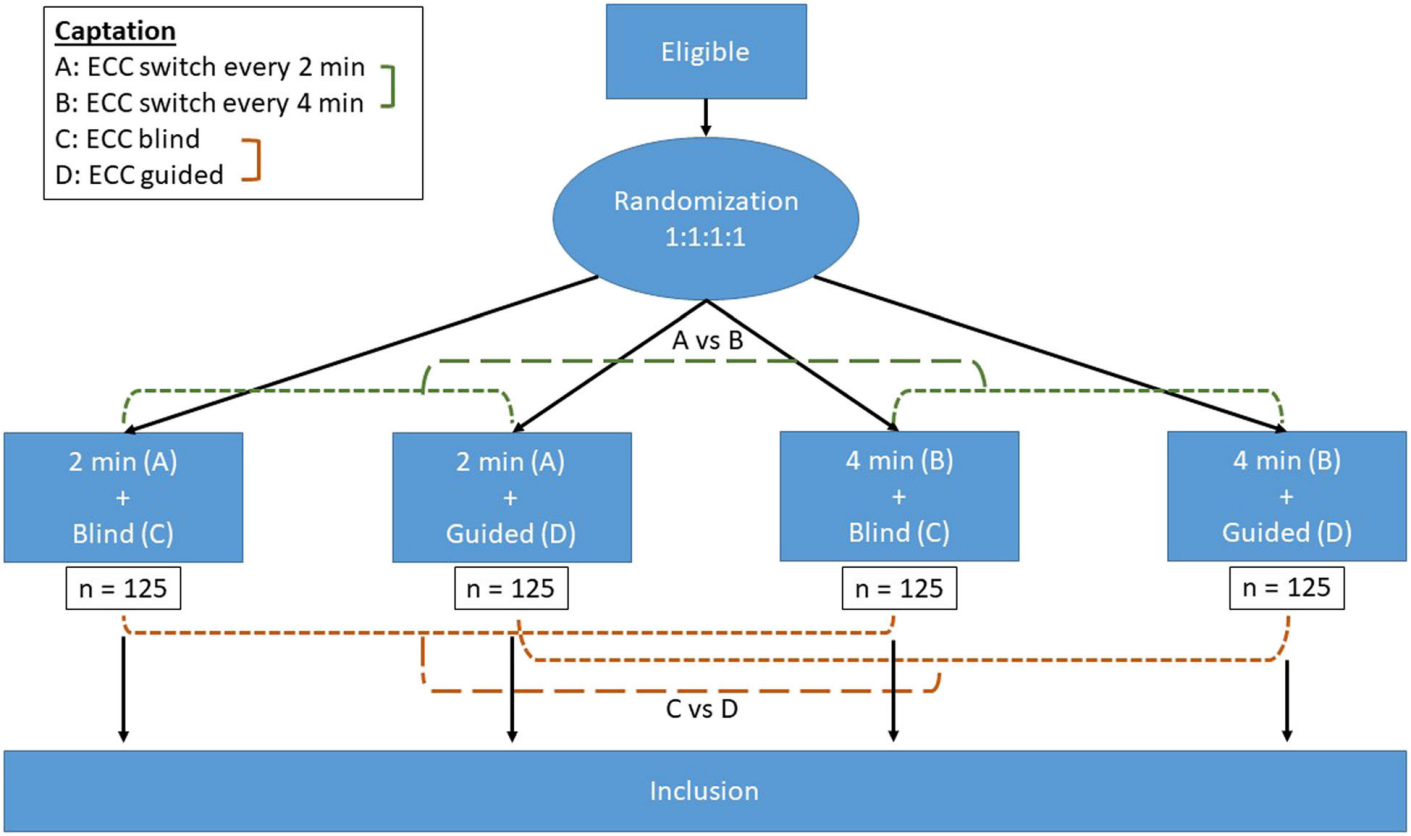
Study design

We propose a randomized, multicenter, open-label study using a 2 × 2 factorial design (Protocol CILICA-HS version 4, June 26, 2019) comparing (i) the rhythm of CC relays every 4 min versus every 2 min on the CCF and (ii) the use of real-time guidance of the CC via a guidance tool (CPRmeter^®^) versus no guidance on the quality of the CC (correct compression score). Emergency prehospital services (Service Mobile d’Urgences et de Réanimation) from 12 hospitals in France will participate. The design of the study is summarized in Fig. 1.

The two main objectives of the factorial plan are as follows:

- To determine whether a CC relay rhythm of 4 min or 2 min is superior in terms of CCF. CCF corresponds to the fraction of CPR time during which there are CC (low-flow) performed by the out-of-hospital resuscitation team on the OHCA patient.
- To determine whether the quality of the CC, measured by CCS, is superior with guidance or without guidance (corresponding to good depth, frequency, and release).

The secondary objectives of this study are to determine whether the impact of the guidance on the quality of the CC and on CCF has an isolated or combined effect on the patient’s outcome: ROSC; survival at day 0, day 1, and day 30 (or earlier intensive care exit); the level of brain injury marker neuron-specific enolase (NSE); and neurological outcome at day 30 (or earlier intensive care exit) (CPC score).

### Selection of participants

Potentially eligible subjects are those with an OHCA for whom an out-of-hospital resuscitation team from an investigative center is involved in the first attempt at resuscitation.

### Inclusion criteria

To be eligible, subjects must meet all defined inclusion criteria:

- Adult
- Victim of an OHCA
- Eligible for inclusion procedure in immediate life emergency
- Affiliated with the social security system

### Noninclusion criteria

A “noninclusion criterion” refers to a criterion identified or known prior to randomization that prevents inclusion in the study. Subjects meeting any of the following noninclusion criteria will not be eligible to participate in the research:

- Not an adult
- More than 6 months pregnant or breastfeeding
- Absence of indication or contraindication for resuscitation: known incurable disease (advanced neurodegenerative diseases, advanced cancers, …), palliative care in progress, a do-not-resuscitate order from the patient or a decision by the medical team not to resuscitate
- Traumatic cardiac arrest
- Impossibility or contraindication to the use of the CC guidance system

### Exclusion criteria

An “exclusion criterion” refers to a secondary finding of a criterion that could not be identified prior to inclusion in the study and that justifies the patient’s exclusion from the study. Subjects meeting any of the following exclusion criteria will not be eligible to participate in the research:

- Medical resuscitation started before inclusion by a noninvestigative team
- An automatic CC device was set up before 5 min of CPR in the protocol
- The CPRmeter^®^ adhesive could not be fixed on the patient’s torso (large breasts, heavy hair, anatomical abnormality, etc.)
- Obvious impairment of the CC quality linked to the use of the CPRmeter^®^
- Discontinuation of CPR before 4 min (excluding ROSC) due to the secondary discovery of the absence of an inclusion criterion or the presence of a noninclusion criterion
- Discovery after the arrival of the medical team of an unidentified noninclusion criterion at the time of randomization

### Blinding

The participants will be in cardiac arrest at the time of their enrollment and at the time of performance of the intervention; they will not be aware of the arm of randomization to which they are initially assigned and will be informed as soon as their clinical status allows it.

The study does not involve a blind setting for the healthcare providers, but outcome assessors and data analysts will be blinded to allocation groups C or D. Data are automatically blinded by the system when they are entered in the online electronic case report form (eCRF). The blinding is not completely possible for groups A and B because the length of the CPR relay differs between the 2 groups and is visible on the data regardless of the allocation masking.

### Randomization, allocation

The block randomization, stratified by trial centers, is performed using a randomization list from a centralized secure online server (internet) 24/7. A backup solution with sealed opaque envelopes will be available in each vehicle participating in the study for situations that do not allow access to the secure randomization server (off-grid area, connection difficulty). There is a stratification of the draw at each center in a 1:1:1:1 distribution: (A + C) 2 min blind, (A + D) 2 min with guidance, (B + C) 4 min blind, and (B + D) 4 min with guidance (Fig. 1).

### Device description

**Fig. 2:**
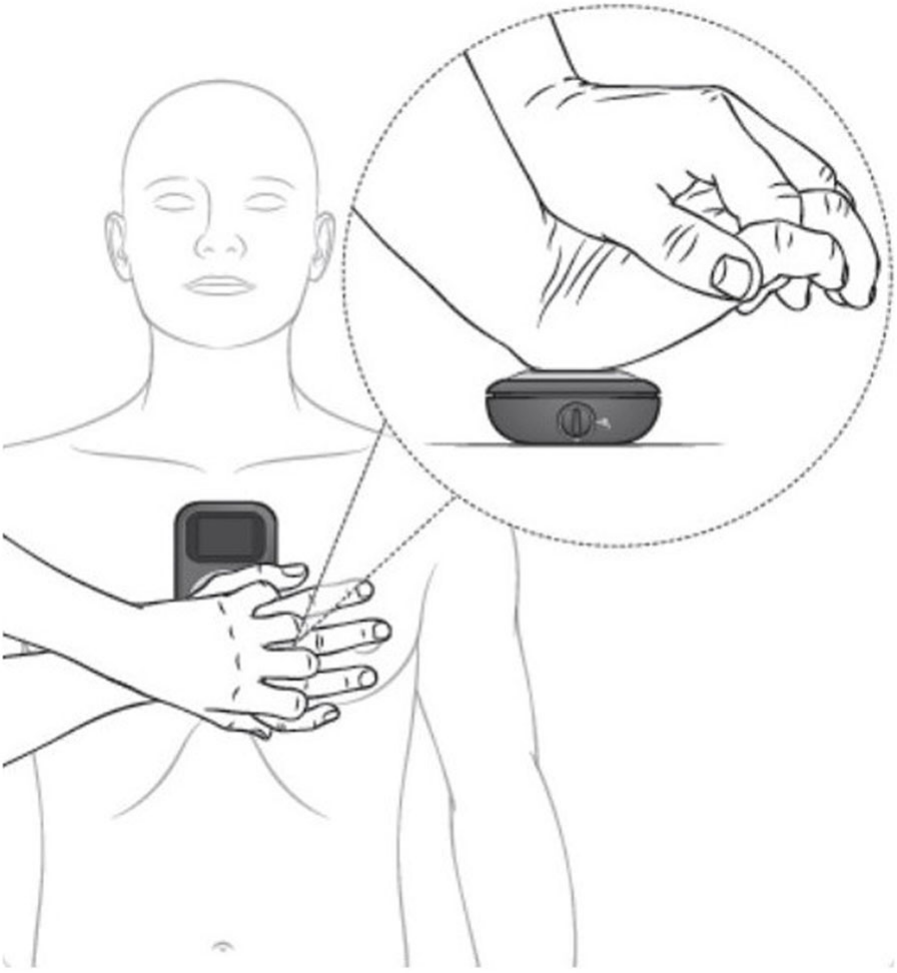
Position and use of CPRmeter^®^ during external chest compression

**Fig. 3:**
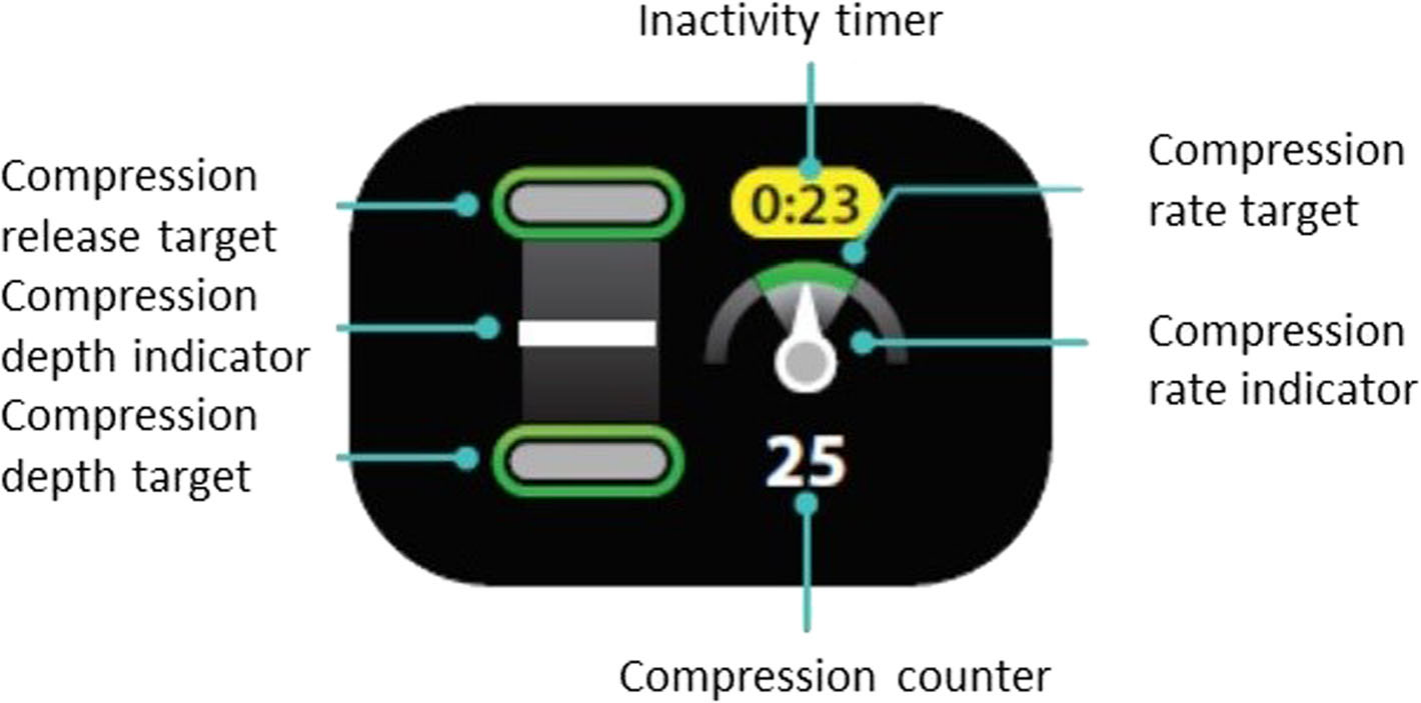
CPRmeter^®^ screen display and feedback provided during external chest compression

The CPRmeter^®^ is a CC guidance device marketed by Laerdal (LAERDAL Medical France, Limonest, France) that provides real-time feedback on CC. The CPRmeter^®^ is placed under the hands of the CC provider on the patient’s chest, where it is secured with a disposable adhesive (Fig. 2). The dimensions of the CPRmeter^®^ are 154 mm × 64 mm × 28 mm, and it weighs 227 g. Feedback data are provided to the user via a 26 mm × 26 mm color screen located above a 100 mm × 55 mm rubber surface for the positioning of the rescuer’s hands. It provides visual feedback to guide the depth, release, and frequency of the CC (Fig. 3). The hand position is represented on the left part of the screen by a white cursor going up and down on a scale according to the CC. Green targets at the top and bottom of the scale illuminate when they are reached by the cursor. In the case of insufficient depth or release CC, yellow arrows appear to indicate the CC modification needed. The CC frequency is shown on the right side of the screen by a needle on a speedometer with a green target area in the middle that illuminates when reached by the needle. The target values are defined by the manufacturer (Laerdal^®^) according to the 2015 recommendations, in effect when the device was designed [9]. The indications provided by the CPRmeter^®^ are visual only and not audio. All data are automatically recorded in real time on the internal memory of the device.

The implementation of the device will be done in collaboration with the Laerdal^®^ Company, which will provide theoretical and practical initial training of investigative teams on the use of CPRmeter^®^ and data recovery before the start of the trial. The presentation and training for the use of the device will be of sufficient duration, and the mastery of the device will be ensured.

### Trial interventions

CA, matching the eligible criteria, managed by an out-of-hospital resuscitation team can be included according to a procedure of immediate vital emergency (article L1122-1-3 of the Code of Public Health) [27]. The randomization is performed by the out-of-hospital resuscitation team’s doctor during the transportation on the spot to know the randomization arm before the arrival at the place of the intervention. The patient is randomized using the online centralized 24/7 server or the backup sealed opaque “off-grid area” emergency randomization envelopes as described in the “Randomization, allocation” section above.

For all groups, the CPRmeter^®^ CC guidance system was positioned on the patient’s chest with a disposable adhesive. The CPRmeter^®^ is always on for all groups. (1) In the CC guidance situation by the CPRmeter^®^ (group D), rescuers have onscreen real-time visual feedback on the quality of the CC performed and indications of corrections to improve the quality, if necessary. (2) In cases of nonguidance of the CC by the CPRmeter^®^ (blind), a specific screen cap is set up on the screen to hide the feedback in the unguided group (group C). The CPRmeter^®^ is always on to record the CC quality data.

The duration or rhythm of a relay is the time during which a rescuer performs a CC before being relayed by another rescuer. This time is 2 min in group A according to the guidelines in effect and 4 min in group B, which is the experimental group.

The CPR of OHCA is therefore normally undertaken according to the guidelines in effect by the out-of-hospital resuscitation team, except for the relay rhythm in group B, which is 4 min. Due to the 2 × 2 factorial design, four situations are possible (Fig. 1):

- CC unguided + relay between 2 rescuers switching at 2 min (C + A)
- CC unguided + relay between 2 rescuers switching at 4 min (C + B)
- CC guided + relay between 2 rescuers switching at 2 min (D + A)
- CC guided + relay between 2 rescuers switching at 4 min (D + B)

Apart from the guidance or blinding of the CC and the duration of the CC relays, the resuscitation is similar to the usual practice of the out-of-hospital resuscitation team and complies with the guidelines in effect [9]. In case of use in relay of the CC of an automated CC device, the patient remains included in the study, the CPRmeter^®^ data (main judgment criteria) are retained, but no more data (secondary criteria) are collected after the implementation of the automated CC device (no blood sampling or CPC score).

Low-flow time is the time during which a CC is performed, generating a minimum cardiac output toward the organs. The no-flow time is the time during which no CC is performed. There is therefore no organ perfusion generated. The no-flow and low-flow times are complementary, and their sum is the patient’s management time. The CCF is the percentage of time during which the patient receives CC over the entire time of rescue (CCF = low-flow time/total time). CC quality data (depth, release, and frequency) as well as time with CC (low-flow) and no CC (no-flow) are automatically collected by the CPRmeter^®^.

The data are collected online via the RéAC registry interface (http://www.registreac.org/), for which a direct data download functionality of the CPRmeter^®^ has been developed in collaboration with Laerdal. The data recorded by the CPRmeter® will therefore be retrieved from the memory after the end of the support upon return to the out-of-hospital resuscitation team’s base and will be transmitted securely—at the same time as those relating to the patient’s care—by Bluetooth connecting the CPRmeter^®^ to the RAC data collection server.

The patient, the family members, or the person of trust will be informed as soon as possible, and their consent (supplementary file) will be sought for the possible continuation of this research (according to article L1122-1-3) [27].

#### Follow-up under study

An NSE measurement will be performed upon the patient’s admission and on day 3 in accordance with the guidelines for the management of postanoxic coma states [28]. Blood samples will be collected into dry blood test tubes at the same time at admission and on day 3 to be sent to the University Hospital of Caen, Center for Biological Resources (CRB InnovaBIO, qualified NFS-96-900). The purpose is to perform a centralized NSE measurement (due to the large variability in the results from one laboratory to another). Samples will be sent within 30 min after sampling to the patient’s hospital center for biological resources (CRB), where they will be centrifuged and frozen at − 80 °C (within a time period of less than 2 h after sampling) and stored in a deep-freeze (− 80 °C) pending repatriation (every 2 months) of all the cryotubes (dry-ice shipping with temperature control) to the Center for Biological Resources (CRB InnovaBIO) of the University Hospital of Caen for analysis. The cryotubes (aliquots needed for dosing) will then be yielded and transferred from the CRB InnovaBIO, in dry ice, to the biochemistry department of the University Hospital of Caen for centralized measurement.

The survival and the CPC score (Table 1) [29] are collected on day 1 by the investigator in coordination with the patient’s unit and are sent to the data collection server.

**Table 1:** Cerebral performance category (CPC) score

Score	Description
1	Good cerebral performance
2	Moderate cerebral disability
3	Severe cerebral disability
4	Coma or vegetative state
5	Death

#### End of study

At day 30 or upon discharge from the intensive care unit, if earlier, or upon the death of the patient, the investigating physician, in coordination with the last unit where the patient was, will collect the following data: survival, number of days of survival in case of death between day 1 and day 30 (or at the exit of resuscitation if earlier), and CPC score [29].

No long-term follow-up is planned.

#### Concomitant care and interventions

No concomitant medications, care, or intervention are prohibited as long as they do not interfere with the rhythm of CC relay or feedback device (CPRmeter^®^) use. The use of an automatic CC device set up before 5 min of CPR in the protocol is an exclusion criterion. If it is set up after 5 min, the collection of the CC data is restricted to those recorded before.

### Outcomes

#### Primary outcomes

Since this is a factorial-design study, there are two primary outcomes:

- The chest compression fraction (CCF) (as a percentage) will be used for the comparison between a 2-min versus 4-min (A vs B) relay of CC. CCF is defined according to Saldanha et al. [30] as a measure of quality of CPR (domain), corresponding to the relative amount of time during which the CC are performed during CPR (specific measurement), formally computed as the ratio of CPR time during which CCs are performed (low-flow) divided by the CPR time performed on the patient by the out-of-hospital resuscitation team. No-flow (no CC performed), low-flow, and CPR times (in seconds) are automatically recorded in real time by the CPRmeter® (metric). The mean CCF will be aggregated for 2 min versus 4 min (A vs B groups) (method of aggregation). The time point of interest for this endpoint is over the duration of CPR (time point).
- The correct compression score (CCS) (as a percentage) will be used for the comparison between real-time feedback guidance by CPRmeter^®^ versus no real-time feedback guidance by CPRmeter^®^ (C vs D). CCF is defined according to Saldanha et al. [30] as a measure of quality of CPR (domain), corresponding to the percentage of CC for which the depth is correct (50 to 60 mm), the frequency is correct (100 to 120/min), and the relaxation is correct (< 2500 g) (specific measurement). The depth (in millimeters), the frequency (number of compressions per minute), and the release (residual strength in grams) of the CC are automatically recorded in real time by the CPRmeter^®^ (metric). The mean CCS will be aggregated for real-time feedback guidance by CPRmeter^®^ versus no real-time feedback guidance by CPRmeter^®^ (C vs D groups) (method of aggregation). The time point of interest for this endpoint is over the duration of CPR (time point).

#### Secondary outcomes

The secondary outcomes are as follows:

- The depth of each CC (in millimeters), recorded continuously by the guidance system (average and percentage correct)
- The frequency of CC (in number of compressions per minute), recorded continuously by the guidance system (average and percentage correct)
- The release of the CC, corresponding to the residual force (in grams), recorded continuously by the guidance system (average and percentage correct)
- The subjective fatigability, assessed by the rescuers who performed CC using the Borg scale (average of the Borg rescuer scale values) [31]
- Time and length of care (in minutes and seconds) based on the following events: CA time, CC start time, the out-of-hospital resuscitation team’s resuscitation start time, and resuscitation end time (ROSC or death of the patient)
- The length (in minutes and seconds) of no-flow and low-flow: no-flow and low-flow times prior to the arrival of the out-of-hospital resuscitation team (declarative) and no-flow and low-flow times during resuscitation by the out-of-hospital resuscitation team (measured by the CC guidance device)
- The rate of return of spontaneous circulation (ROSC)
- The survival rate at hospital admission
- The value of NSE at admission and day 3 [28]
- The survival rate on day 1 and day 30 (or resuscitation output if earlier)
- Cerebral performance category score (CPC) at day 30 or intensive care discharge [29]

The study will be conducted within the framework of the French national network of the RéAC Cardiac Arrest Registry (http://www.registreac.org/).

### Data collection

Data will be collected using international Utstein-style guidelines on reports for OHCA and using the automatic data registration of the CPRmeter^®^ device. Data from the CPRmeter^®^ will be uploaded to the section of the French national network of the RéAC Cardiac Arrest Registry (http://www.registreac.org/) dedicated to the study. All other data will be collected by doctors in charge of the patient and entered into an eCRF of the dedicated section of the RéAC Cardiac Arrest Registry. Clinical research assistants (locally and in the promoting center) will help and monitor the data collection and the eCRF. The data input is checked by computer.

Apart from the CC quality data report by the CPRmeter^®^, data collection will be performed as follows:

#### At day 0

- Demographic characteristics: identity, social security number, intervention, street address, age (year), sex (male/female), weight (estimated, in kilograms), height (estimated, in meters)
- Patient history: medical history (cardiovascular, respiratory, diabetes, end of life, others)
- History of the disease: presumed etiology of CA (cardiac, neurological, respiratory, asphyxiation, poisoning, drowning, unknown, others), durations of no-flow and low-flow periods before medical resuscitation (minutes), existence of witnesses, resuscitation maneuvers undertaken before the arrival of professional rescuers (CC, defibrillation, ventilation)
- History of the advanced CPR carried out by the out-of-hospital resuscitation team:
  - Duration of low-flow, no-flow, and advanced resuscitation (minutes, obtained by CPRmeter^®^ recording)
  - Quality of the CC and its components (frequency in CC per minute, depth in millimeters, and release in grams obtained by the CPRmeter^®^ recording)
  - EtCO_2_ values (mmHg) 1 min (± 20 s) after intubation, the highest obtained during CC (before ROSC) and at the end of resuscitation (1 min ± 20 s after the ROSC or during the decision to end resuscitation) [32, 33]
  - External electrical shocks (number, intensity in joules)
  - Initial rhythm upon the out-of-hospital resuscitation team arrival (asystole, ventricular fibrillation, ventricular tachycardia without pulse, electromechanical dissociation)
  - Numbers and total doses of vasopressor amines and antiarrhythmic administration (milligrams)
  - ROSC or death

- Survival at hospital admission

#### At hospital discharge or at day 30

- Values of the NSE performed upon the patient’s admission and on day 3 (in micrograms per liter) [28]
- Survival on day 1 and day 30 (or at the intensive care unit (ICU) discharge if earlier)
- Length of stay in the ICU (days)
- Neurological state assessment by the CPC and any sequelae on day 1 and at ICU discharge or on day 30 [29]

### Data management

Trained research staff (clinical research assistants) at each center will collect data using an online national secure database dedicated to cardiac arrest data collection (RéAC; http://www.registreac.org/). The two different eCRF pages for prehospital and intensive care will be completed separately. Data on CC quality from the CPRmeter^®^ will be uploaded with the clinical data from the prehospital period. Deidentified completed data will be sent to the principal investigator at Caen University Hospital. Furthermore, data will be monitored by a data manager. The clinical research manager and two clinical research assistants from the steering committee will be available to help and monitor the data collection and management.

### Data monitoring committee

The data monitoring committee from the national cardiac arrest registry RéAC will ensure the first level of independent data monitoring. A specific-to-the-study data monitoring committee composed of a senior data manager and an assistant data manager (independent from the primary sponsor and the steering committee) will regularly control the maintenance of the informatic system, check the quality of the data entered, and ensure the proper functioning of the automatic data entry control system.

### Safety and potential adverse events

The study implies few changes from OHCA guidelines as real-time feedback is not currently strongly recommended but a possibility, and the time for CPR relay is based on little concrete data [15]. However, feedback tool use has been reported to be safe [20, 34]. In the context of an OHCA, no significant adverse event related to the study is expected. Adverse events can be reported through eCRF, email, and phone calls to the promoter. Serious adverse events will be investigated, and reports will be provided directly to the safety monitoring committee. If they occur, adverse events will be reported in the publication.

### Safety monitoring

A trio of independent experts not involved in the study (safety monitoring committee) will meet when 250 patients have been included to review the monitored data on ROSC and survival on day 0, day 1, and day 30 according to the randomized group. The investigator can request an extra meeting at any time in case of new data in the literature or if an event occurs in the study requiring the safety monitoring committee’s advice.

### Steering committee

A steering committee composed of the principal investigator, a statistician, a clinical research manager, and two clinical research assistants will be in charge of the presentation, the implementation and follow-up of the study at the different participating centers, and the overall management of the study (coordination of the data management team and safety committee).

### Auditing

Research assistants from the steering committee will conduct at least one onsite monitoring visit per year over the course of the study at 100% of the recruiting sites (with repeat visits to sites where performance is a concern). The primary objectives during the onsite visits are to educate, support, and solve problems. At the start of the trial, the monitors will conduct a tutorial on the procedure to extract data from the CPRmeter^®^ and on the online data entry system. The investigators will practice so that the monitors can confirm that the investigators are proficient in all aspects of data extraction and entry.

### Schedule of data collection

Intervention assignment will be performed before starting the trial. If patients survive their CA, they will be followed up for 1 month after enrollment. The schedule of data collection is summarized in Table 2.

**Table 2:**
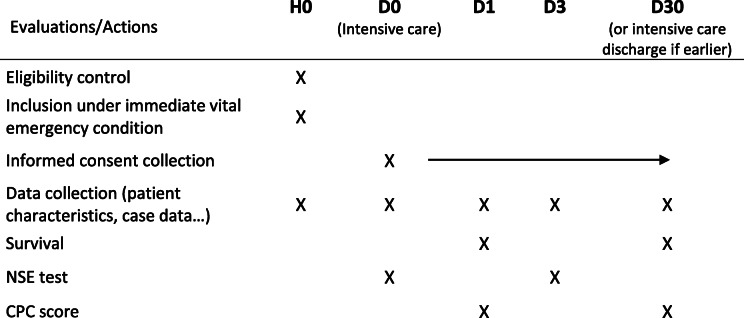
Schedule of data collection

### Trial registration

The trial was registered on 28 January 2019 at http://www.clinicaltrials.gov under number NCT03817892 (https://clinicaltrials.gov/ct2/show/NCT03817892?term=buleon&draw=2&rank=1).

### Statistical analyses

The main analysis population will be defined as all randomized patients for whom CPR has been engaged, according to a modified intent-to-treat principle. Randomized patients who do not fulfill the inclusion criteria, die, or have a ROSC at prehospital team arrival will be excluded from the main modified intent-to-treat principle analysis. We plan to conduct a pure intent-to-treat analysis in which these excluded patients and patients with missing data will have their outcome imputed by multiple imputation process. This analysis will test the effect of these exclusions on trial outcomes. The statistical analysis plan will follow the recommendations of the tests with a factorial plan described in the CONSORT statement [35], namely, (i) the verification of the absence of interaction between the 2 interventions tested (here on different criteria of judgment) and (ii) a separate analysis by type of multivariate intervention that will systematically include the effect of the untested intervention. Regarding the main criterion of judgment, the average percentage of CCF for comparison of A versus B and the average percentage of CCS for comparison C versus D, the two groups will be compared in terms of superiority of the 4-min group (B) and the guidance group (D) by a multivariate generalized linear regression model including the randomization group as an explanatory variable as well as the other intervention received as a covariable of the model and possibly the interaction of the 2 interventions if it is significant (Student’s test for independent series).

The other criterion (secondary outcomes) will be compared between the groups using the appropriate tests and in an exploratory manner. For example, the qualitative variables will be compared between the groups with the *χ*^2^ test, the quantitative variables by Student’s *t* test, and the survival by the log-rank test.

All confidence intervals of the parameters to be estimated will be established at 5% risk (95% confidence interval). No interim analysis will be performed for primary outcomes (CCF and CCS). The significance level is set at 0.05. The analysis will be performed in SAS software v9.4 (SAS Institute NC, Cary).

### Determination of sample size

We estimated the calculation of the number of subjects required in this trial using a factorial design for the comparison of 2 min versus 4 min (A vs B) on the average percentage of CCS.

With an average CCF of 70% [36] in the control group (group A, 2 min) and a 5% improvement in the experimental group (group B, 4 min), a power of 90%, a two-tailed alpha risk of 5% bilateral, and a standard deviation of ± 17%, 243 subjects per group are required to compare the effect of 2 min versus 4 min (A vs B) on the average percentage of CCF.

Regarding the guiding hypothesis, with a difference of 15% in the CCS between the guided group (D) and the blinded group (C), a standard deviation of ± 36% (12), alpha 5%, and beta 10%, the number of subjects needed is lower (122 per group).

We plan to include 500 patients according to the sample size needed for hypothesis A vs B (243 per group), which is higher than that needed for hypothesis C vs D (122 per group).

## Discussion

The study will contribute to the field of literature on the impact of real-time feedback on CC quality in practical conditions of OHCA resuscitation. No definitive position on the benefit of real-time feedback on CC quality has been assessed [9, 20]. This topic is complicated and has many possible confounding factors and biases. A definitive answer will probably come from a meta-analysis or a large-scale study. It seems to us that this study can be one of the small steps toward a conclusion.

The study will also provide insight into the feasibility of extending the switch time duration between two rescuers from the currently recommended 2 to 4 min. Beyond feasibility, it will provide clues on the effect of an extension of the switch time on the CC quality. With the two groups, guided and blinded to real-time feedback, it will also determine whether an extension of the switch time provides a CC quality as efficient as the current 2 min regardless of real-time feedback, only with real-time feedback or whether CC quality decays even with real-time feedback. Even if the extension of the switch time does not have a positive effect on CC quality, we will have concrete data on its effect on the chest compression fraction time. Since this has been highlighted as a determining element of the quality of resuscitation, it will provide an interesting perspective for future research and care in CA. As an anticipated limitation, we know that the sample size—designed to answer our questions—will probably be insufficient to provide significant data on mortality and morbidity. However, we believe this study may help provide a clearer view of some important aspects of the management of OHCA and may open new opportunities for further research.

### Trial status

The trial is ongoing, and patient recruitment is active. The first patient was included on December 6, 2019. The recruitment is estimated to be completed by November 30, 2021 (protocol version 4 from June 26, 2019).

## Supplementary information

**Additional file 1.** Patient informed consent to continue a study.

**Additional file 2.** World Health Organization Trial Registration.

## Data Availability

Only the steering committee has access to the full trial dataset to ensure the overall results. Site investigators may access their site or the full dataset if a formal request describing their plans is approved by the steering committee. The trial protocol is available online on clinicaltrial.gov. Three years after the end of the collection of the data, once all publications related to the study will be done, the full dataset will be accessible with the steering committee’s approval upon formal request describing the purpose.

## Abbreviations

CA: Cardiac arrest
CC: Chest compression
CCF: Chest compression fraction
CCS: Correct compression score
CPC: Cerebral performance category
CPR: Cardiopulmonary resuscitation
eCRF: Electronic case report form
EtCO_2_: End-tidal carbon dioxide (CO_2_)
ICU: Intensive care unit
NSE: Neuron-specific enolase
OHCA: Out-of-hospital cardiac arrest
ROSC: Return of spontaneous circulation

## References

[1] Escutnaire J, Genin M, Babykina E, Dumont C, Javaudin F, Baert V (2018). Traumatic cardiac arrest is associated with lower survival rate vs. medical cardiac arrest - results from the French national registry. Resuscitation.

[2] Benjamin EJ, Muntner P, Alonso A, Bittencourt MS, Callaway CW, Carson AP (2019). Heart disease and stroke statistics-2019 update: a report from the American Heart Association. Circulation..

[3] Hawkes C, Booth S, Ji C, Brace-McDonnell SJ, Whittington A, Mapstone J (2017). Epidemiology and outcomes from out-of-hospital cardiac arrests in England. Resuscitation..

[4] The Cardiac Arrest Registry to Enhance Survival (CARES). Demographic and survival characteristics of OHCA [Internet]. 2019 Apr. Available from: https://mycares.net/sitepages/uploads/2019/2018%20Non-Traumatic%20National%20Summary%20Report.pdf. Accessed 10 Apr 2020.

[5] Gräsner J-T, Bossaert L (2013). Epidemiology and management of cardiac arrest: what registries are revealing. Best Pract Res Clin Anaesthesiol.

[6] Gräsner J-T, Lefering R, Koster RW, Masterson S, Böttiger BW, Herlitz J (2016). EuReCa ONE-27 Nations, ONE Europe, ONE Registry: a prospective one month analysis of out-of-hospital cardiac arrest outcomes in 27 countries in Europe. Resuscitation..

[7] International Liaison Committee on Resuscitation (2005). 2005 International Consensus on cardiopulmonary resuscitation and emergency cardiovascular care science with treatment recommendations. Part 4: advanced life support. Resuscitation.

[8] Nolan JP, Soar J, Zideman DA, Biarent D, Bossaert LL, Deakin C (2010). European resuscitation council guidelines for resuscitation 2010: section 1. Executive summary. Resuscitation.

[9] Monsieurs KG, Nolan JP, Bossaert LL, Greif R, Maconochie IK, Nikolaou NI (2015). European resuscitation council guidelines for resuscitation 2015: section 1. Executive summary. Resuscitation.

[10] Christenson J, Andrusiek D, Everson-Stewart S, Kudenchuk P, Hostler D, Powell J (2009). Chest compression fraction determines survival in patients with out-of-hospital ventricular fibrillation. Circulation..

[11] Wik L, Olsen J-A, Persse D, Sterz F, Lozano M, Brouwer MA (2016). Why do some studies find that CPR fraction is not a predictor of survival?. Resuscitation..

[12] Berg RA, Sanders AB, Kern KB, Hilwig RW, Heidenreich JW, Porter ME (2001). Adverse hemodynamic effects of interrupting chest compressions for rescue breathing during cardiopulmonary resuscitation for ventricular fibrillation cardiac arrest. Circulation..

[13] Cunningham LM, Mattu A, O’Connor RE, Brady WJ (2012). Cardiopulmonary resuscitation for cardiac arrest: the importance of uninterrupted chest compressions in cardiac arrest resuscitation. Am J Emerg Med.

[14] Kilgannon JH, Kirchhoff M, Pierce L, Aunchman N, Trzeciak S, Roberts BW (2017). Association between chest compression rates and clinical outcomes following in-hospital cardiac arrest at an academic tertiary hospital. Resuscitation..

[15] Kleinman ME, Brennan EE, Goldberger ZD, Swor RA, Terry M, Bobrow BJ (2015). Part 5: adult basic life support and cardiopulmonary resuscitation quality: 2015 American Heart Association guidelines update for cardiopulmonary resuscitation and emergency cardiovascular care. Circulation..

[16] Perkins GD, Jacobs IG, Nadkarni VM, Berg RA, Bhanji F, Biarent D (2015). Cardiac arrest and cardiopulmonary resuscitation outcome reports: update of the Utstein resuscitation registry templates for out-of-hospital cardiac arrest: a statement for healthcare professionals from a Task Force of the International Liaison Committee on Resuscitation (American Heart Association, European Resuscitation Council, Australian and New Zealand Council on Resuscitation, Heart and Stroke Foundation of Canada, InterAmerican Heart Foundation, Resuscitation Council of Southern Africa, Resuscitation Council of Asia); and the American Heart Association Emergency Cardiovascular Care Committee and the Council on Cardiopulmonary, Critical Care, Perioperative and Resuscitation. Resuscitation.

[17] Cheskes S, Schmicker RH, Rea T, Morrison LJ, Grunau B, Drennan IR (2017). The association between AHA CPR quality guideline compliance and clinical outcomes from out-of-hospital cardiac arrest. Resuscitation..

[18] Yannopoulos D, Aufderheide TP, Abella BS, Duval S, Frascone RJ, Goodloe JM (2015). Quality of CPR: an important effect modifier in cardiac arrest clinical outcomes and intervention effectiveness trials. Resuscitation..

[19] Wang PL, Brooks SC (2018). Mechanical versus manual chest compressions for cardiac arrest. Cochrane Database Syst Rev.

[20] Hostler D, Everson-Stewart S, Rea TD, Stiell IG, Callaway CW, Kudenchuk PJ (2011). Effect of real-time feedback during cardiopulmonary resuscitation outside hospital: prospective, cluster-randomised trial. BMJ..

[21] Buléon C, Delaunay J, Parienti J-J, Halbout L, Arrot X, Gérard J-L (2016). Impact of a feedback device on chest compression quality during extended manikin CPR: a randomized crossover study. Am J Emerg Med.

[22] Goodloe JM, Idris AH (2017). Metrics save lives: value and hurdles faced. Curr Opin Crit Care.

[23] Lin S, Scales DC (2016). Cardiopulmonary resuscitation quality and beyond: the need to improve real-time feedback and physiologic monitoring. Crit Care.

[24] Cheskes S, Byers A, Zhan C, Verbeek PR, Ko D, Drennan IR (2017). CPR quality during out-of-hospital cardiac arrest transport. Resuscitation..

[25] Wallace SK, Abella BS, Becker LB (2013). Quantifying the effect of cardiopulmonary resuscitation quality on cardiac arrest outcome: a systematic review and meta-analysis. Circ Cardiovasc Qual Outcomes.

[26] Reynolds JC, Raffay V, Lang E, Morley PT, Nation K (2015). When should chest compressions be paused to analyze the cardiac rhythm? A systematic review and meta-analysis. Resuscitation..

[27] Legifrance. Code de la santé publique - Article L1122-1-3 [Internet]. Code de la santé publique Jun 16, 2016. Available from: https://www.legifrance.gouv.fr/affichCodeArticle.do?idArticle=LEGIARTI000032722920&cidTexte=LEGITEXT000006072665&dateTexte=20161231. Accessed 10 Apr 2020.

[28] Vondrakova D, Kruger A, Janotka M, Malek F, Dudkova V, Neuzil P (2017). Association of neuron-specific enolase values with outcomes in cardiac arrest survivors is dependent on the time of sample collection. Crit Care.

[29] Wijdicks EFM, Hijdra A, Young GB, Bassetti CL, Wiebe S (2006). Quality Standards Subcommittee of the American Academy of Neurology. Practice parameter: prediction of outcome in comatose survivors after cardiopulmonary resuscitation (an evidence-based review): report of the Quality Standards Subcommittee of the American Academy of Neurology. Neurology.

[30] Saldanha IJ, Dickersin K, Wang X, Li T (2014). Outcomes in Cochrane systematic reviews addressing four common eye conditions: an evaluation of completeness and comparability. PLoS One.

[31] Borg GA (1982). Psychophysical bases of perceived exertion. Med Sci Sports Exerc.

[32] Murphy RA, Bobrow BJ, Spaite DW, Hu C, McDannold R, Vadeboncoeur TF (2016). Association between prehospital CPR quality and end-tidal carbon dioxide levels in out-of-hospital cardiac arrest. Prehosp Emerg Care.

[33] Pantazopoulos C, Xanthos T, Pantazopoulos I, Papalois A, Kouskouni E, Iacovidou N (2015). A review of carbon dioxide monitoring during adult cardiopulmonary resuscitation. Heart Lung Circ.

[34] Kirkbright S, Finn J, Tohira H, Bremner A, Jacobs I, Celenza A (2014). Audiovisual feedback device use by health care professionals during CPR: a systematic review and meta-analysis of randomised and non-randomised trials. Resuscitation.

[35] Schultz K, Griffiths J, Lacasse M (2015). The application of entrustable professional activities to inform competency decisions in a family medicine residency program. Acad Med.

[36] Vaillancourt C, Everson-Stewart S, Christenson J, Andrusiek D, Powell J, Nichol G (2011). The impact of increased chest compression fraction on return of spontaneous circulation for out-of-hospital cardiac arrest patients not in ventricular fibrillation. Resuscitation..

