# Supplementary material for: Impacts of chest compression cycle length and real-time feedback with a CPRmeter® on chest compression quality in out-of-hospital cardiac arrest: study protocol for a multicenter randomized controlled factorial plan trial
Source: Trials. 2020 Jul 8;21:627. doi: 10.1186/s13063-020-04536-3 (PMC7346361; doi:10.1186/s13063-020-04536-3)
Supplement: Supplementary file 1 — Additional file 1. Patient informed consent to continue a study. [file 13063_2020_4536_MOESM1_ESM.docx]

| **PATIENT INFORMED CONSENT TO CONTINUE A STUDY** |
| --- |

**Title of the research**

**IMPACTS OF CHEST COMPRESSION CYCLE LENGTH AND REAL-TIME FEEDBACK WITH A CPRMETER® ON CHEST COMPRESSION QUALITY:**

**C**OMPRESSION **I**S **L**IFE **I**N **C**ARDIAC **A**RREST **-** **H**UMAN **S**TUDY (CILICA-HS)

| **PROMOTOR:**  CHU de CAEN Normandie  Avenue de la côte de nacre  CS 30001 - 14033 CAEN  🕾 : 02.31.06.57.81  N° d’enregistrement: 2018-A02000-55 | **INVESTIGATOR COORDINATOR:**  Docteur Clément BULEON  Service de Réanimation Chirurgicale et d’Anesthésie  CHU de Caen Normandie  Avenue de la Côte de Nacre  CS 30001 - 14033 CAEN  🕾 : 02.31.06.43.80 |
| --- | --- |

**With the understanding that:**

- I have been included in this study under a procedure in an immediate life-threatening emergency as defined by law.
- I have been clearly informed of the purpose and duration, as well as the foreseeable benefits and risks of this clinical research, and I have received satisfactory answers to all the questions I have asked.
- I understand that in order to be able to participate in this research, I must be affiliated to a social security system or be entitled to social insurance and I confirm that this is the case.
- I have had sufficient time for reflection between the information and the signing of the consent.
- I may at any time decide to interrupt my participation in this study without justification and without incurring any liability or prejudice as a result. This will in no way affect the quality of care that will be provided.
- This consent does not relieve the sponsor and the investigators of all their responsibilities.
- My right to access and rectify my personal data, as provided for by the law n° 2018-493 of 20 June 2018 on the protection of personal data, may be exercised at any time through the doctor who is following me for this study.
- The study has received all the regulatory authorizations for its implementation.
- A dated and signed copy of this document will be given to me.

**I agree that**:

- In a free and voluntary way, I continue my participation in intervention protocol entitled "Impacts of chest compression cycle length and real-time feedback with a CPRmeter^®^ on chest compression quality ".
- The documents in my medical file that relate to the study may be accessible to those in charge of the study and possibly to the health authorities. With the exception of these people, who will treat the information in the strictest respect of medical confidentiality, my anonymity will be preserved.
- The data recorded in the course of this study will be processed by computer.

| **I allow that:** | |
| --- | --- |
| - My biological samples are kept at the C.R.B Innova BIO of the Caen Normandy University Hospital and used for research on my pathology or related pathologies. Genetic analyses are carried out and that the results of these analyses can be the subject of computerized processing by the team responsible for the study. |  |

| ***To be completed by the patient:***  ***NAME****___________________________________________________*  ***FIRST NAME****_______________________________________________* | Date: __________________________  Signature: |
| --- | --- |

| ***To be completed by the investigator-doctor:***  ***NAME****__________________________________________________*  ***FIRST NAME****______________________________________________* | Date: __________________________  Signature: |
| --- | --- |

*One copy of this document will be given to you, one copy will be kept by the investigator.*
