# Supplementary material for: Impacts of chest compression cycle length and real-time feedback with a CPRmeter® on chest compression quality in out-of-hospital cardiac arrest: study protocol for a multicenter randomized controlled factorial plan trial
Source: Trials. 2020 Jul 8;21:627. doi: 10.1186/s13063-020-04536-3 (PMC7346361; doi:10.1186/s13063-020-04536-3)
Supplement: Supplementary file 2 — Additional file 2. World Health Organization Trial Registration. [file 13063_2020_4536_MOESM2_ESM.docx]

| **WORL HEALTH ORGANIZATION TRIAL REGISTRATION** |
| --- |

**Title of the research**

**IMPACTS OF CHEST COMPRESSION CYCLE LENGTH AND REAL-TIME FEEDBACK WITH A CPRMETER® ON CHEST COMPRESSION QUALITY:**

**C**OMPRESSION **I**S **L**IFE **I**N **C**ARDIAC **A**RREST **-** **H**UMAN **S**TUDY (CILICA-HS

| **Data category** | **Information** |
| --- | --- |
| Primary registry and trial identifying number | ClinicalTrials.gov NCT03817892 |
| Date of registration in primary registry | 28 January 2019 |
| Secondary identifying numbers | N° ID-RCB : 2018-A02000-55  N° CHU : 17-248 |
| Source(s) of monetary or material support | Direction Générale de l’Offre de Soins (DGOS) |
| Primary sponsor | Direction Générale de l’Offre de Soins (DGOS) |
| Secondary sponsor(s) | The University Hospital of Caen |
| Contact for public queries | Direction de la Recherche Clinique et de l’Innovation, CHU de Caen Normandie, Avenue Cote de Nacre 14000 Caen, France  +33(0)231065781 ; [drci-sec@chu-caen.fr](mailto:drci-sec@chu-caen.fr) |
| Contact for scientific queries | Dr Clément Buléon, MD, MSc CHU de Caen Normandie, Avenue Cote de Nacre 14000 Caen, France |
| Public title | Impacts of chest compression cycle length and real-time feedback with a CPRmeter on chest compression quality in out-of-hospital cardiac arrest (CILICA-HS) |
| Scientific title | Impacts of chest compression cycle length and real-time feedback with a CPRmeter on chest compression quality in out-of-hospital cardiac arrest: study protocol for a multicenter randomized controlled factorial plan trial |
| Countries of recruitment | France |
| Health condition(s) or problem(s) studied | cardiac arrest |
| Intervention(s) | Active comparator: (i) 4 min relay of chest compression and (ii) guidance of the CPR performance by a CPRmeter.  Non-active comparator: (i) 2 min relay of chest compression and (ii) no guidance of the CPR performance by a CPRmeter. |
| Key inclusion and exclusion criteria | Ages eligible for study: ≥18 years Sexes eligible for study: both Accepts healthy volunteers: no **Inclusion criteria:** adult patient (≥ 18 years), victim of an out-hospital cardiac arrest, eligible for inclusion procedure in immediate life emergency, affiliated with the social security system  **Noninclusion criteria:** Not an adult, more than 6 months pregnant or breastfeeding, absence of indication or contraindication for resuscitation (known incurable disease, palliative care in progress, do-not-resuscitate order from the patient or a decision of the medical team not to resuscitate), traumatic cardiac arrest, impossibility or contraindication to the use of the CC guidance system. **Exclusion criteria:** Medical resuscitation started before inclusion by a non-investigative team, an automatic chest compression device set up before 5 minutes of CPR in the protocol, the CPRmeter® adhesive could not be fixed on the patient's torso (large breasts, heavy hair, anatomical abnormality, etc.), obvious impairment of the CC quality linked to the use of the CPRmeter®, discontinuation of CPR before 4 minutes (excluding return of spontaneous circulation) due to the secondary discovery of the absence of an inclusion criterion or the presence of a noninclusion criterion, discovery after the arrival of the medical team of an unidentified noninclusion criterion at the time of randomization. |
| Study type | Factorial plan 2x2 Multicenter  Randomized  Masking: blind (subject, outcomes assessor) |
| Date of first enrolment | December 2019 |
| Target sample size | 500 |
| Recruitment status | Recruiting |
| Primary outcome(s) | (i) To determine whether a chest compression relay rhythm of 4 minutes or 2 minutes is superior in terms of chest compression fraction. Chest compression fraction corresponds to the fraction of CPR time during which there is chest compression (low-flow) performed by the out-of-hospital resuscitation team on the cardiac arrest patient.  (ii) To determine whether the quality of the chest compression, measured by compression score, with guidance or without guidance is superior (corresponding to good depth, frequency and release). |
| Key secondary outcomes | The secondary objectives of this study are to determine whether the impact of the guidance on the quality of the chest compression and on chest compression fraction has an isolated or combined effect on the patient's outcome: return of spontaneous circulation; survival at day 0, day 1 and day 30 (or earlier intensive care exit); the level of brain injury marker neuron-specific enolase ; and neurological outcome at day 30 (or earlier intensive care exit) (CPC score). |
